# Preparation and characterization of geraniol nanoemulsions and its antibacterial activity

**DOI:** 10.3389/fmicb.2022.1080300

**Published:** 2022-11-29

**Authors:** Xiaolin Feng, Kexin Feng, Qinhua Zheng, Weijian Tan, Wenting Zhong, Caiyu Liao, Yuntong Liu, Shangjian Li, Wenzhong Hu

**Affiliations:** ^1^College of Pharmacy and Food Science, Zhuhai College of Science and Technology, Zhuhai, China; ^2^College of Life Science, Jilin University, Changchun, China

**Keywords:** geraniol, nanoemulsions, characterization, stability, antibacterial activity

## Abstract

Geraniol nanoemulsions (G-NE) based on Tween 80 and medium chain triglyceride (MCT) as surfactant and co-surfactant, respectively, has been prepared by the spontaneous emulsification method. Its physical and chemical properties such as mean particle size, zeta potential, PDI, pH, viscosity, contact angle, appearance morphology, and stability (storage stability, thermal stability, centrifugal properties, acid-base stability, and freeze-thaw properties) of the droplet were analyzed. The results showed that the mean particle size of G-NE was 90.33 ± 5.23 nm, the PDI was 0.058 ± 0.0007, the zeta potential was −17.95 ± 5.85 mV and the encapsulation efficiency was >90%. The produced G-NE has been demonstrated to be fairly stable in long-term storage at 4°C, pH = 5 and high-speed centrifuges. Moreover, G-NE had a significant inhibition effect on *Staphylococcus aureus*, *Escherichia coli*, *Salmonella typhimurium* and *Listeria monocytogenes* (*p < 0.05*). The bacterial inhibition rates of G-NE at a concentration of 1 MIC were 48, 99, 71.73, and 99% after 12 h of action against these four foodborne pathogenic bacteria, respectively. Therefore, the results obtained indicated that nanoemulsification enhanced the stability and antibacterial activity of geraniol to some extent, which will promote the utilization of geraniol in food preservation.

## Introduction

Food safety has become one of the major public health issues in the world (Shaker et al., 2022). Currently, most food products (bread, dairy products, meat, fruits and vegetables, etc.) are heavily contaminated due to the presence of various pathogenic microorganisms and their associated toxins, leading to serious human illness and death. To control the growth of bacteria and fungi, various synthetic preservatives are commonly applied. However, the use of these preservatives may cause negative effects such as food safety, microbial resistance, and environmental contamination (Calvo et al., 2017). Therefore, it is very important to find a safe, effective, and non-polluting natural preservative for the storage and preservation of food products.

Plant essential oils (EOs) and their components are receiving increasing attention in the commercial food sector for their unique aroma, flavor, and antimicrobial properties without affecting the sensory and nutritional properties of the food (Maurya et al., 2021a; Li et al., 2022). EOs are secondary metabolites of aromatic plants and are listed by the FDA under the Generally Recognized as Safe (GRAS) label (Ju et al., 2018).

**FIGURE 1:**
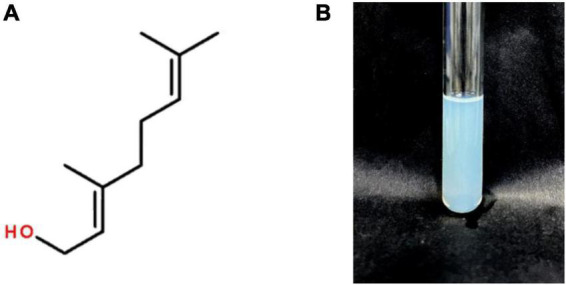
Structure of geraniol **(A)** and Appearance of the prepared G-NE **(B)**.

Geraniol is an acyclic monoterpene alcohol contained in the volatile oils of various plants with the chemical formula C_10_H_18_O (Figure 1A). Geraniol is naturally found in *Pelargonium hortorum*, *Rosa rugosa*, *Cymbopogon citratus [DC] Stapf*, *Elsholtzia ciliata (THunb.) Hyland.* and other plants. It is widely used in pharmaceutical, tobacco and food ingredient fields. Studies have shown that geraniol has insecticidal (Niculau et al., 2013), antibacterial (Miladinovic et al., 2014; Bhattamisra et al., 2018), preventive and inhibitory effects on cancer cell proliferation (Carnesecchi et al., 2001; Jin et al., 2013; Ortiz et al., 2022), anti-diabetic (Babukumar et al., 2017), and anti-oxidative stress (El Azab et al., 2022; Nisar et al., 2022). However, the application of geraniol is greatly limited by its natural properties, such as high volatility, pungent odor, chemical instability and low water solubility (Chen and Viljoen, 2010). To overcome these drawbacks, they need to be modified or encapsulated using different methods or materials.

Nanotechnology will become one of the core technologies in the field of fine food processing in the 21st century. For nanoemulsions, some countries have conducted in-depth research in cosmetics and new drug preparation, especially in Europe, Japan and the United States. Nanoemulsions are kinetically stable colloidal systems with small droplet sizes ranging from 50 to 200 nm (Aswathanarayan and Vittal, 2019). The small size of droplets in nanoemulsions means that they usually have better gravitational separation, flocculation and agglomeration stability than macroemulsions (Liu et al., 2019). Therefore, the preparation of geraniol into nanoemulsions is expected to be a safe, stable and biodegradable formulation for food packaging or drug delivery. However, its physical properties and functional activity should be thoroughly evaluated before use in food preservation or drug formulations. In this study, geraniol nanoemulsions (G-NE) were prepared by using a self-emulsification method to encapsulate geraniol. The main objectives of this study were: (i) to obtain more comprehensive information on the physicochemical properties of G-NE using different analytical techniques for characterization; (ii) to evaluate the variable stability and in vitro antimicrobial activity of G-NE.

## Materials and methods

### Materials

Geraniol (Shanghai Aladdin Biochemical Technology Co., China), Tween 80 (Shanghai Yuanye Biotechnology Co., Ltd., China), medium chain triglyceride (Shanghai Yuanye Biotechnology Co., Ltd., China), medium chain triglyceride (Shanghai Yuanye Biotechnology Co., Ltd., China), sodium citrate (Shanghai Yuanye Biotechnology Co., Ltd. Ltd., China), sterile TTC solution (0.5%) (Guangdong Huan Kai Biotechnology Co., China), *Staphylococcus aureus* (GDMCC 112442), *Escherichia coli* (GDMCC 1.173), *Salmonella typhimurium* (GDMCC 11442), *Listeria monocytogenes* (GDMCC 12408) were purchased from Guangdong Microbial Strain Collection Center, China.

### Preparation of nanoemulsions

The nanoemulsions were prepared with reference to the method of Chang et al. with some minor modifications (Chang et al., 2013). The oil phase (4 wt% geraniol + 6 wt% MCT) and surfactant (15 wt% Tween 80) were combined first, and the mixture was then thoroughly blended in a magnetic stirrer (25°C, 800 rpm/min) before being gradually titrated at a rate of 1 ml/min into the 75 wt% aqueous phases (5 mM citrate buffer, pH 5.5).

### Characterization of the nanoemulsion

#### Determination of the basic properties of geraniol nanoemulsion

The test was performed by stain diffusion at 25°C with the addition of 1 mg/ml of water-soluble methylene blue and oil-soluble Sudan IV solutions to G-NE. If the diffusion rate of methylene blue base was larger than that of Sudan red, it was an O/W type; otherwise, it was a W/O type (He et al., 2017).

#### Mean particle size, polydispersity index, particle size distribution and zeta potential

The mean particle size, polydispersity index (PDI), particle size distribution and zeta potential of the samples were analyzed at 25°C with a laser particle size/potential analyzer (Delsa Nano C, Beckman Coulter, USA). The results of the analysis for each recorded measurement are the average of 3 scans.

#### Physicochemical properties of geraniol nanoemulsion

The viscosity of G-NE was measured at 25°C using a digital viscometer (SNB-2, Shanghai Jingtian Electronic Instruments Co., Ltd., China).

The pH of G-NE was measured at 25°C using a handheld pH meter (OHAUS Corporation, USA).

Measure the color of G-NE at 25°C using a colorimeter (CR-10 PLUS, Konica Minolta Sensing Co., Ltd., Osaka, Japan), determine L*, a* and b* values, and calculate the whiteness index (WI).


$$\text{WI}=100-{\left[{\left(100-L\right)}^{2}+{\text{a}}^{2}+{\text{b}}^{2}\right]}^{\frac{1}{2}}$$

The surface tension of G-NE was measured at 25°C using a contact angle meter (SL200L2, Shanghai Soren Information Technology Co., Ltd., China).

The contact angle of G-NE was measured on a smooth glass surface using a contact angle measuring instrument (SL200L2, Shanghai Soren Information Technology Co., Ltd., China).

#### Transmission electron microscope

The copper mesh containing the support film was placed on a wax plate, 5 μl of G-NE was added dropwise on the film and dried naturally, then 5 μl of 2% phosphotungstic acid was added dropwise and the excess liquid was blotted up with filter paper, and the morphology of the microemulsion was observed under a transmission electron microscope (JEM2100, Nippon Electron Co., Ltd., Japan) after drying naturally.

#### Determination of geraniol content and encapsulation efficiency

Take 500 μl of G-NE into a 10 ml volumetric flask, add appropriate amount of anhydrous ethanol to dissolve, then ultrasonic for 15 min, and continue to fix the volume with anhydrous ethanol to the scale line. Take 0.1 ml of the ultrasonicated solution into a 10 ml volumetric flask and dilute to the scale with anhydrous ethanol. The absorbance was measured by UV spectrophotometer (UV-2600i, Shimadzu, Japan), and the content of total active substance (*W*_*t*_) was calculated according to the standard curve and the relationship between dilution times.

Take 500 μl G-NE and centrifuge it in an ultrafiltration tube at 10,000 r/min for 15 min to obtain the clarified centrifugal solution. The filtrate was aspirated, dissolved with anhydrous ethanol, and fixed into a 10 ml volumetric flask. The concentration of free active ingredient (*W*_*f*_) was determined using the standard curve and dilution multiple relationship after the absorbance was measured using a UV spectrophotometer. The encapsulation efficiency (EE) was calculated according to the formula.


$$\text{EE}=\frac{\left({\text{W}}_{t}-{\text{W}}_{f}\right)}{{\text{W}}_{t}}\times 100\%$$

### Stability

The prepared G-NE was stored under sealed and light-proof conditions at 4 and 25°C for 28 days. Samples of the prepared G-NE were taken at 0, 7, 14, 21, and 28 days to determine the storage stability of G-NE.

The prepared G-NE was centrifuged at 4,000, 6,000, and 8,000 rpm/min for 20 min to examine its centrifugation stability.

To simulate pasteurization during the processing of food, the fabricated G-NE has been heated in a water bath at 60, 70, 80, and 90°C for 30 min to determine its thermal stability.

The prepared G-NE was adjusted with hydrochloric acid (1 mol/ml) and sodium hydroxide (1 mol/ml) to adjust the PH to 2.0, 3.0, 4.0, 5.0, 6.0, 7.0, 8.0, and 9.0. The combination was then sealed and kept at 4°C in the dark for an hour to assess G-NE’s acid-base stability.

The prepared G-NE was subjected to freeze-thaw cycles at 37 and −20°C alternately every 24 h under sealed, light-proof storage conditions for 1 week to measure the freeze-thaw of G-NE.

The appearance, particle size, and geraniol content variations of G-NE were used as evaluation criteria in all stability trials.

### Antimicrobial activity

#### Measurement of bacterial inhibition circle

The inhibition effect of G-NE on four foodborne pathogenic microorganisms, *Staphylococcus aureus (S. aureus)*, *Escherichia coli (E. coli)*, *Salmonella typhimurium (S. typhimurium)* and *Listeria monocytogenes (L. monocytogenes)*, was determined by the punching method. 100 μl of bacterial broth with a concentration of 1 × 10^6^ CFU/ml was spread on NA medium, 4 wells were punched on the medium, and then 90 μl of geraniol (G), G-NE, emulsion without geraniol (C-NE), and blank control (C) were added to the wells, respectively. The treated petri dishes were sealed and incubated at 37°C for 24 h. The size of the BIC is measured and photographed (Kotan et al., 2007). Every experiment’s material and tool are autoclaved, and the ultra-clean bench is used for all operations.

#### Minimum inhibitory concentration

The minimum inhibitory concentration (MIC) of G-NE was measured by micro-broth dilution method by referring to the experimental method of Miladinović et al. with slight modifications (Miladinovic et al., 2014). 100 μl of LB medium mixed with sterile TTC solution (0.5%) was added to a sterile 96-well plate, 100 μl of G-NE was added to the first well, and then the mixture was diluted sequentially to the twelfth well by twofold dilution method, and finally 100 μl of bacterial solution (1 × 10^6^ CFU/ml) was added to each well. Each row of 12 wells is a group of experimental groups, the blank group is added to the medium, and the control group is added to C-NE, and the procedure is repeated three times. The MIC of the experimental bacteria treated with geraniol was the same as above.

#### Growth curve

Referring to the experimental method of Amrutha et al. with slight modifications (Amrutha et al., 2017), 200 μl of LB medium was added to a 96-well plate, followed by additions of 0.5 MIC, 1 MIC, and 2 MIC concentrations of G-NE and a thorough shaking. 20 μl of bacterial solution (1 × 10^6^ CFU/ml) was added to each well and the culture was incubated at 37°C, the OD value at 600 nm was measured and recorded in every 2 h. Three biological replicates were performed for each group.

### Statistical analysis

All experiments were performed two to three times using freshly prepared samples, and results are reported as the mean and standard deviation of these measurements. Statistical analysis was performed using Origin 2021, Graphpad Prism 8 and SPSS. To compare differences between multiple groups, analysis of variance (ANOVA) was performed on the data. *p < 0.05* were considered statistically significant.

## Results

### Characterization of the nanoemulsion

#### Determination of the basic properties of geraniol nanoemulsion

The prepared G-NE is slightly turbid with blue opalescence as shown in Figure 1B. After adding methylene blue dye and Sudan IV dye dropwise to the emulsion, the diffusion rate of methylene blue dye in the emulsion was significantly faster than that of Sudan IV dye, so the emulsion prepared in this test can be judged as O/W type nanoemulsion.

#### Basic characteristics of geraniol nanoemulsion

**FIGURE 2:**
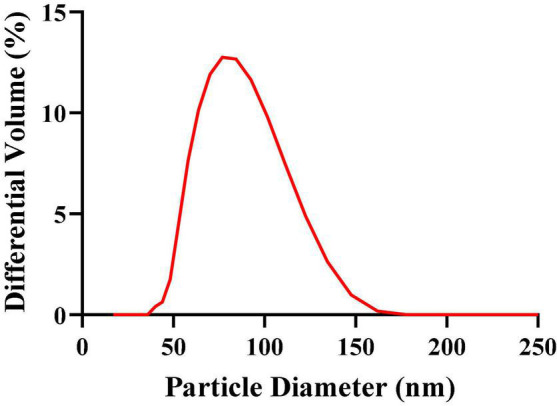
Particle size distribution of G-NE.

The mean particle size, PDI, zeta potential, and particle size distribution are very important indicators of nanoemulsions, which can describe the size, stability, uniformity, and dispersion of nanoemulsions. The greater the zeta potential, the greater the mutual repulsion between particles, and the better the stability of the dispersion system. The experimental results obtained by a laser particle size/potential analyzer (Delsa Nano C) are shown in Table 1, where the mean particle size of G-NE is 90.33 ± 5.23 nm, the PDI is 0.058 ± 0.0007, and the zeta potential is − 17.95 ± 5.85 mV. The particle size distribution as shown in Figure 2, the peak width is very narrow, indicating that it has uniform droplet size. The results show that the system is stable, the G-NE mean particle size is small, the droplet dispersion is good, the size is uniform, and there is essentially no aggregation.

**TABLE 1:** Basic characteristics of G-NE.

Sample	Mean particle size (nm)	Polydispersity index (PDI)	Zeta potential (mV)
G-NE	90.33 ± 5.23	0.058 ± 0.0007	−17.95 ± 5.85

#### Physicochemical properties of geraniol nanoemulsion

The results are shown in Table 2, where obtained G-NE viscosity was 5.14 ± 0.35 mPa⋅s, pH was 5.8 ± 0.2, whiteness index was 31.78 ± 0.26, contact angle was 8.99 ± 0.03°, and surface tension was 21.43 ± 0.11 mN/m. The results are consistent with the mean particle size since the lower viscosity suggests that the surfactant molecules move more quickly and easily, resulting in smaller particles. According to Jimenez et al. (2018), WI values close to 100 correspond to opaque/white systems. Nanoemulsions are slightly turbid systems and the appearance of nanoemulsions is largely influenced by the concentration and diameter of the oil droplets, therefore, as the diameter of the oil droplets increases, light scattering is stronger and the nanoemulsion tends to be opaque. In general, lower surface tension formulations typically give good spreading capabilities (Teixeira et al., 2017). The interfacial tension is one of the elements influencing how quickly Oswald ripens, and the lower it is, the more it delays the start of ripening. However, for an actual influence, the interfacial tension needs to be decreased by several orders of magnitude (Tadros et al., 2004).

**TABLE 2:** Physicochemical properties of geraniol nanoemulsion (G-NE).

Sample	Viscosity (mPa ⋅ s)	Whiteness index	pH	Contact angle (о)	Surface tension (mN/m)
G-NE	5.14 ± 0.35	31.78 ± 0.26	5.8 ± 0.2	8.99 ± 0.03	21.43 ± 0.11

#### Morphology of geraniol nanoemulsion droplets

**FIGURE 3:**
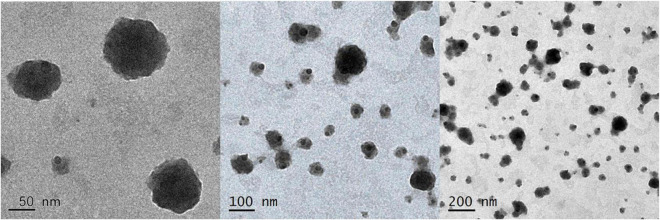
Particle form of G-NE.

According to Figure 3, prepared G-NE droplets are well dispersed as they are evenly distributed, spherical, and of a uniform size when viewed by TEM.

#### Determination of geraniol content and encapsulation efficiency

**FIGURE 4:**
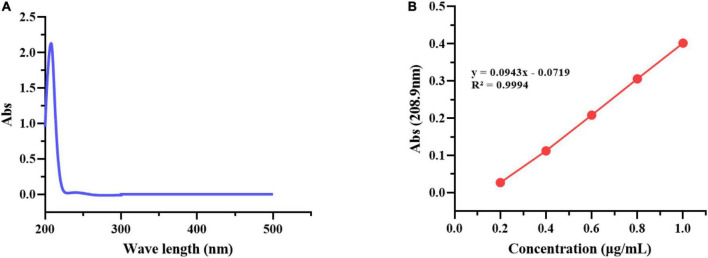
UV absorption spectrum of geraniol **(A)** and Standard curve of geraniol **(B)**.

As Figure 4A shows the UV absorption spectrum of geraniol, it has good UV absorption, with the maximum absorption at 208.9 nm. As Figure 4B shows the standard curve of geraniol, the regression equation was obtained: *y* = 0.0943*x* − 0.0719, and the correlation coefficient *R*^2^ = 0.9994, the regression straight line is a good fit to the observed values. The geraniol content and encapsulation efficiency (EE) of G-NE were 2.32 ± 0.13 and 93.22 ± 1.53%, respectively, which are substantial compared to most reported values (Zhang and Zhong, 2020; Zhuang et al., 2022).

### Stability

#### Storage stability

**FIGURE 5:**
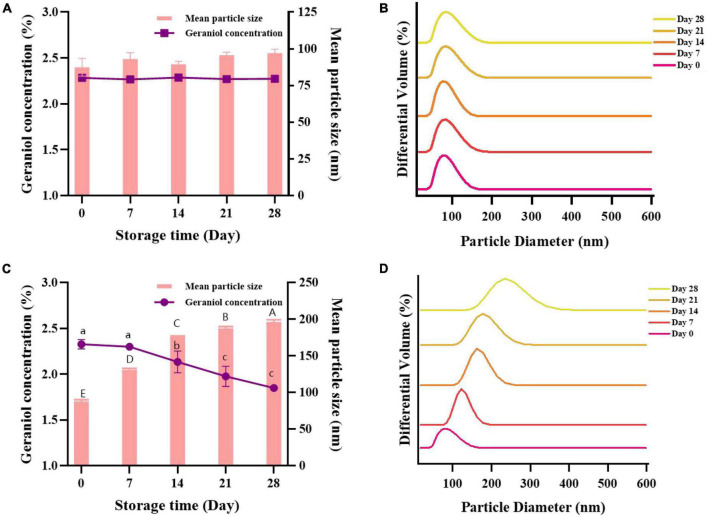
Effect of long-term storage at 4°C **(A,B)** and 25°C **(C,D)** on the mean particle size, geraniol content, and particle size distribution of G-NE. (Different capital (lowercase) letters indicate significant intra-group variability, *p < 0.05*; identical capital (lowercase) letters indicate significant inter-group variability, *p > 0.05*).

The stability of nanoemulsions can be determined in large part by the size and composition of their particles. The storage stability experiment provides a reference for the storage conditions and expiration date of microemulsions. G-NE was stored at 4 and 25°C for 28 days, and the changes of mean particle size and geraniol content were tested to evaluate its physicochemical stability. As shown in Figures 5A–D, with the increase of storage time, there was no significant difference in the mean particle size and geraniol content of G-NE after 28 days of storage at 4°C, with uniform particle size distribution and good storage stability. The mean particle size of G-NE rose from 89 to 197 nm after 28 days of storage at 25°C, its geraniol concentration declined from 2.30 to 1.85%, and the particle size distribution was not concentrated and the emulsion was stratified, which indicated its poor stability in storage. The analysis may be due to the slow droplet motion at 4°C and the nanoscale particle size which makes the effect of gravity on it greatly weakened (Li et al., 2013), the interaction forces between the components of the system reach equilibrium and form a stable dispersion system, so that instability phenomena such as creaming, precipitation, flocculation, agglomeration, and Ostwald maturation leading to phase separation are less likely to occur during the storage process (McClements, 2012). In conclusion, the nanoemulsions stored at a constant temperature of 4°C were able to maintain better stability and were more conducive to the storage of G-NE.

#### Centrifugation stability

The effects of rotational speed on the mean particle size, geraniol content, and particle size distribution of G-NE are shown in Table 3. All samples showed no delamination, no significant difference in content and particle size, and concentrated particle size distribution after centrifugation at different speeds for 20 min, which suggested that the G-NE gained had relatively favorable centrifugal stability. It might be related to the nanoparticle size and the corresponding viscosity of the microemulsion system (Grit and Crommelin, 1993).

**TABLE 3:** Effect of different rotational speeds on the mean particle size and geraniol content of G-NE.

Rotational speed (rpm/min)	Geraniol concentration (%)	Mean particle size (nm)
4,000	2.39 ± 0.02%^a^	88 ± 1^A^
6,000	2.42 ± 0.03%^a^	88 ± 2^A^
8,000	2.34 ± 0.05%^a^	89.3 ± 3.3^A^

Different capital (lowercase) letters indicate significant intra-group variability, *p* < *0.05*; identical capital (lowercase) letters indicate significant inter-group variability, *p* > *0.05*.

#### Thermal stability

**FIGURE 6:**
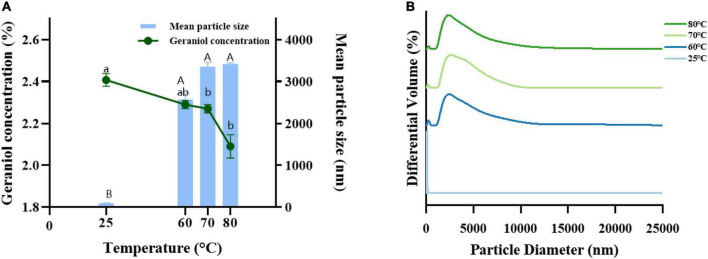
Effect of heating to 60°C, 70°C, and 80°C on the mean particle size **(A)**, geraniol content **(A)** and particle size distribution **(B)** of G-NE. (Different capital (lowercase) letters indicate significant intra-group variability, *p < 0.05*; identical capital (lowercase) letters indicate significant inter-group variability, *p > 0.05*).

G-NE was heated at different temperatures for 30 min to test the stability of microemulsions at different temperatures and to provide a basis for its practical application. Figure 6 shows that after 30 min of heating G-NE at 60, 70, and 80°C, the emulsion turned milky white and oil-water separation took place. The geraniol content of G-NE dropped as the mean particle size increased and the particle size distribution became more uneven. At this point, the nanoemulsion’s structure was irreparably damaged, and even after cooling to ambient temperature, it was impossible to return it to its previous state. G-NE stratified and the top layer turned milky white and solidified after 30 min of 90°C heating. This indicates that G-NE might be poorly stabilized at high temperatures and the low temperature environment is more suitable for storing it.

The significant increase in average droplet size can be attributed to agglomeration and/or Ostwald maturation phenomenon (Chang and McClements, 2014). Initially, nanoemulsions consist of small droplets (nanoscale). However, these nanoemulsions are quite unstable and lead to significant droplet growth at high temperatures, and these large new droplets may aggregate. Droplet flocculation occurs whenever the net attractive force of the dispersed phase exceeds its interfacial tension (Ben Jema et al., 2018). Therefore, it is advisable to store these formulated food-grade nanoemulsions at low or ambient temperatures to reduce the risk of nanoemulsion degradation (Falleh et al., 2021).

#### Acid-base stability

**FIGURE 7:**
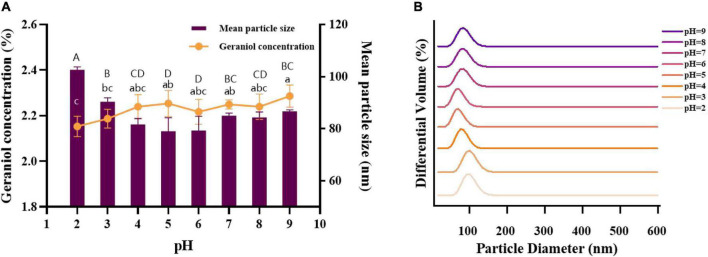
Effect of different pH on the mean particle size **(A)**, geraniol content **(A)** and particle size distribution **(B)** of G-NE. (Different capital (lowercase) letters indicate significant intra-group variability, *p < 0.05*; identical capital (lowercase) letters indicate significant inter-group variability, *p > 0.05*).

The stability of nanoemulsions under acid-base conditions is also one of the important indicators to be considered because of the wide pH range of beverages and foods. An acid-base buffer was prepared instead of deionized water to test the tolerance of the microemulsions under different pH conditions of acidity and alkalinity, and the results are shown in Figure 7. The pH increased from 2.0 to 5.0, the mean particle size of G-NE decreased, and the geraniol content increased. pH increased from 5.0 to 9.0, the mean particle size of G-NE increased and there was no significant difference in the geraniol content. pH values in the range of 2.0–9.0 showed a concentrated particle size distribution and no stratification was observed. When pH = 5.0, the mean particle size of G-NE was the smallest and the geraniol content was relatively high. In summary, G-NE was quite stable in the pH range of 2.0–9.0 and showed the best physical stability at pH = 5.0 (Qian et al., 2012).

#### Freeze-thaw stability

**FIGURE 8:**
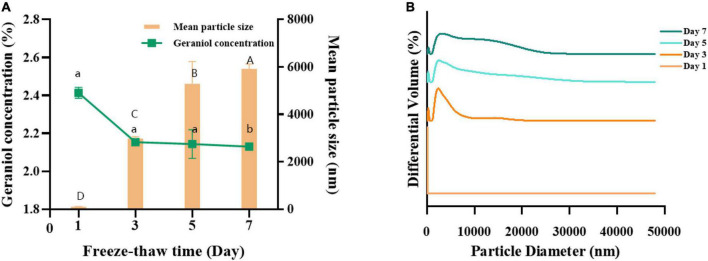
Effect of freeze-thaw cycles on the mean particle size **(A)**, geraniol content **(A)** and particle size distribution **(B)** of G-NE. (Different capital (lowercase) letters indicate significant intra-group variability, *p < 0.05*; identical capital (lowercase) letters indicate significant inter-group variability, *p > 0.05*).

Freezing is a common method used in food processing to retain the quality and nutritional properties of foods and effectively reduce the growth and propagation of microorganisms, so the freeze-thaw stability of the emulsions needs to be investigated. The effect of freeze-thaw cycle treatment on G-NE is shown in Figure 8. After three freeze-thaw cycles, the mean particle size of G-NE increased from 90.33 ± 5.23 to 17,266.7 ± 636.67 nm, the particle size distribution was not concentrated, the content of geraniol decreased from 2.41 to 2.13%, and oil-water separation occurred, which may be due to the crystallization during the thawing process and resulted in the rupture of the interfacial film, the droplet aggregation and oil-water separation. In summary, the freeze-thaw stability of G-NE is poor (Khan et al., 2015).

### Antimicrobial activity

#### Bacterial inhibition circle

**FIGURE 9:**
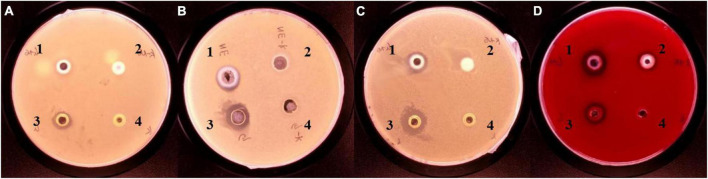
Inhibitory effects of different treatments on *Staphylococcus aureus* **(A)**, *Escherichia coli* **(B)**, *Salmonella typhimurium* **(C)**, and *L. monocytogenes* **(D)**. [1: G-NE, 2: emulsion without geraniol (C–NE), 3: equal amount of free geraniol (G), 4: blank control (C)].

The inhibition ability of G-NE was determined by punching method, and it is shown in Figure 9 that G-NE has growth inhibition effect on *S. aureus*, *E. coli*, *S. typhimurium* and *L. monocytogenes*. The results of the BIC diameter are shown in Table 4, which showed that the BIC size of *S. aureus* was 9.71 ± 0.14 mm, *E. coli* was 11.20 ± 0.24 mm, *S. typhimurium* was 11.97 ± 0.58 mm, and *L. monocytogenes* was 15.80 ± 0.53 mm. The experiment proved that geraniol retained its excellent inhibition ability after being encapsulated into G-NE.

**TABLE 4:** Inhibition effect of different treatments on the four model bacteria.

Type of	Diameter of BIC of different			
bacteria	system categories (mm)			
	
	G-NE	C-NE	G	C
*Staphylococcus aureus*	9.71 ± 0.14^b^	8.24 ± 0.27^c^	12.63 ± 0.07^a^	–
*Escherichia coli*	11.20 ± 0.24^a^	8.56 ± 0.09^b^	11.09 ± 0.64^a^	–
*Salmonella typhimurium*	11.97 ± 0.58^a^	7.65 ± 0.27^c^	15.43 ± 0.33^b^	–
*Listeria monocytogenes*	15.80 ± 0.53^a^	11.44 ± 0.21^c^	12.97 ± 0.31^b^	–

The same letter indicates no significant difference between groups, different letters indicate significant difference between groups, *p* < *0.05*; –: There is no BIC.

#### Minimum inhibitory concentration

The MIC of G-NE was determined as shown in Table 5. The MIC of G-NE was 7.81 μl/ml for *S. aureus*, 3.91 μl/ml for *E. coli*, 3.91 μl/ml for *S. typhimurium*, and 7.81 μl/ml for *L. monocytogenes*. It can be seen that the preparation of geraniol into G-NE still has antibacterial effect and has lower MIC compared with pure geraniol.

**TABLE 5:** Minimum inhibitory concentration (MIC) of the four model bacteria by different treatments.

	MIC for different system			
Type of bacteria	categories (μl/ml)			
	
	G-NE	C-NE	G	C
*Staphylococcus aureus*	7.8125^c^	>125^a^	15.625^b^	>125^a^
*Escherichia coli*	3.90625^a^	>125^a^	7.8125^b^	>125^a^
*Salmonella typhimurium*	3.90625^a^	>125^a^	7.8125^b^	>125^a^
*Listeria monocytogenes*	7.8125^b^	>125^a^	7.8125^b^	>125^a^

The same letter indicates no significant difference between groups, different letters indicate significant difference between groups, *p* < *0.05*.

#### Growth curve

**FIGURE 10:**
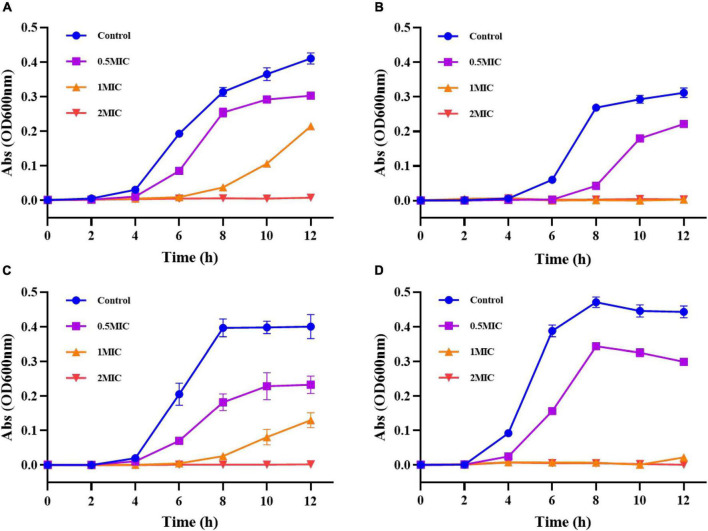
Growth curves of *Staphylococcus aureus* **(A)**, *Escherichia coli* **(B)**, *Salmonella typhimurium* **(C)**, and *Listeria monocytogenes* **(D)** after treatment with different concentrations of G-NE.

As shown in Figure 10A, the results showed that *S. aureus* in the control group reached the exponential growth phase at 4 h and entered the stable phase at 12 h. After treatment with 0.5 MIC of G-NE, *S. aureus* entered the exponential growth phase after 4 h and entered the stable phase after 10 h. The growth curve was 2 h earlier than that of the control group; G-NE with 1 MIC entered the exponential growth period after 8 h and did not reach the stable phase at 12 h. The absorbance of 2 MIC did not change during 12 h, which indicated that *S.aureus* was very sensitive to 2 MIC concentration; different concentrations of G-NE inhibited *S.aureus* during 12 h.

As shown in Figure 10B, the growth of *E. coli* in control group reached the exponential growth period at 6 h of incubation and entered the stabilization period at 12 h. *E. coli* entered the exponential growth period at 8 h after G-NE treatment with 0.5 MIC and did not enter the stabilization period at 12 h. The absorbance of *E. coli* in 12 h after G-NE treatment with 1 MIC and 2 MIC did not change. This indicates that *E. coli* is very sensitive to the concentration of 1 MIC and 2 MIC; different concentrations of G-NE inhibited *E. coli* during 12 h.

*Salmonella typhimurium* in the control group reached the exponential growth phase at 4 h of incubation and entered the stable phase at 8 h demonstrated in Figure 10C. After G-NE treatment with 0.5 MIC, *S. typhimurium* entered the exponential growth phase after 4 h and entered the stable phase after 10 h. The growth curve lagged 2 h behind that of the control group; after G-NE treatment with 1 MIC, *S. typhimurium* entered the exponential growth phase after 8 h and had not reached the stable phase at 12 h. *S. typhimurium* entered the exponential growth period after 8 h and did not reach the stable phase at 12 h. The absorbance of *S. typhimurium* did not change in 12 h after G-NE treatment with 2 MIC, which indicated that *S. typhimurium* was very sensitive at 2 MIC concentration; different concentrations of G-NE inhibited the growth of *S. typhimurium* during 12 h.

As shown in Figure 10D, *L. monocytogenes* in the control group reached the exponential growth phase at 4 h of incubation and entered the stable phase at 8 h. After G-NE treatment with 0.5 MIC, *L. monocytogenes* entered the exponential growth phase after 4 h and entered the stable phase after 8 h. The absorbance of *L. monocytogenes* did not change within 12 h, which indicated that *L. monocytogenes* was very sensitive at 1 MIC and 2 MIC concentrations; G-NE at 1 MIC and 2 MIC concentrations had an inhibitory effect on *L. monocytogenes* within 12 h.

The antibacterial activity of essential oil nanoemulsions depends mainly on the concentration of essential oil in the emulsion, the particle size, and the release rate of the essential oil from the emulsion. As expected, higher G-NE concentrations correspond to better antibacterial activity (Noori et al., 2018). The sustained release of geraniol from the nanoemulsions over time prolonged the action of the essential oils on microorganisms, and the experiments demonstrated that G-NE maintained its inhibitory effect on four food-borne pathogenic bacteria within 12 h.

## Discussion

Since there has been a focus on green, natural foods in recent years, EOs have gained popularity as natural antibacterial agents and food preservatives with a significant market potential. However, Eos’ use in food preservation has been constrained by their low water solubility and high volatility (Chouhan et al., 2017; Rao et al., 2019). Therefore, it is essential to address their shortcomings. There has been an increasing interest in the application of nanoemulsions in recent years, and the application research covers pharmaceutical, cosmetic and food fields (Teo et al., 2010; Marzuki et al., 2019; Chaurasiya et al., 2021; Sneha and Kumar, 2022). The development of desirable and kinetically stable nanoemulsions of essential oil-based antimicrobials frustrating the accompanying limitations and enhances water solubility for improved application in food (Pavoni et al., 2020).

Currently, nanoemulsions are prepared by high-energy emulsification (high-pressure homogenization, microjet and ultrasonic shear) and low-energy emulsification (phase transition temperature, reverse-phase emulsification and self-emulsification) (Silva et al., 2012). High-energy emulsification methods are energy intensive and thus have limited applications, while low-energy emulsification methods have significant potential for bulk preparation. In this study, we prepared and characterized geraniol nanoemulsions by self-emulsification, and the results of laser particle size/potential analyzer showed that G-NE is an O/W nanoemulsion with small particle size, good dispersion, and concentrated particle size distribution. TEM analysis of the morphology of G-NE reveals isolated particles that do not aggregate. They are comparable to measurements of the average droplet size made using the Delsa Nano C (Verma et al., 2021). The benefits of nanoemulsions include small particle size and high dispersion. These properties allow them to defy gravity through Brownian motion, preventing precipitation during storage and ensuring the homogeneity of the system (Marhamati et al., 2021; Siraj et al., 2021). Nanoemulsions, on the other hand, are thermodynamically unstable, and the smaller the droplet size, the higher the interfacial energy, the more likely it is that Ostwald ripening will occur, and the simpler it will be to transfer fluid from small to large droplets, which ultimately causes the emulsion to coarsen (McClements, 2012). Pilong et al. showed that nanoemulsions containing clove essential oil increased in particle size when stored under high temperature conditions (Pilong et al., 2022). This study’s preparation of G-NE demonstrated thermodynamic instability as well, which is consistent with the findings of Pilong et al. However, G-NE has strong kinetic stability and somewhat mitigates the instability of geraniol by itself.

**FIGURE 11:**
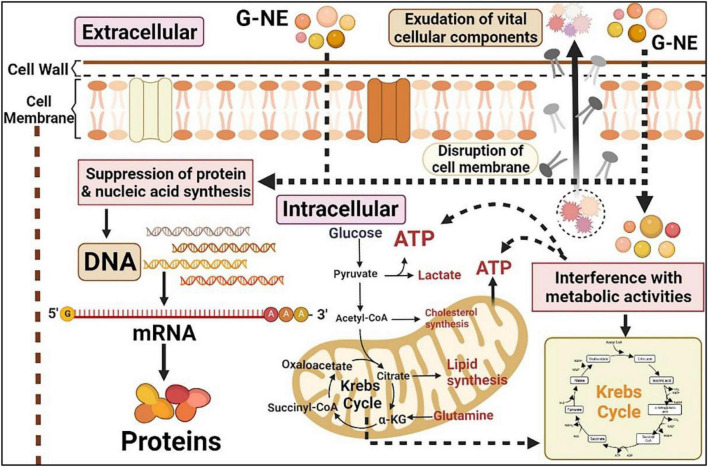
Effects of G-NE on bacteria (The figure was created employing online software named www.biorender.com).

The most important way in which essential oils exert their antibacterial activity is by disrupting the microbial cell membrane structure (Hassan et al., 2015; Mutlu-Ingok et al., 2020). We speculate G-NE can enter the phospholipid bilayer structure of the cell membrane. The geraniol in the nanoemulsion then binds to the protein sites of the cell membrane, promoting changes in the organization and structure of the cell membrane, increasing its permeability and causing the leakage of important cell contents, resulting in cell lysis and death (Falleh et al., 2020). In addition, the incorporation of G-NE into cells may causes the following effects on the cells (Figure 11): (1) inhibition of cellular uptake of nutrients; (2) alteration of intracellular ATP content or reduction of ATP synthase activity; (3) inhibition of electron transfer in the respiratory chain; and (4) inhibition of protein and nucleic acid synthesis (Donsi and Ferrari, 2016). In this study, we found that geraniol was encapsulated into G-NE still retained the excellent antibacterial ability of geraniol, and for some bacteria G-NE showed more excellent antibacterial ability than geraniol. The outcomes of this study are compatible with the findings of Gharenaghadeh et al., who encapsulated Salvia multicaulis essential oil and shown that the antibacterial activity of Salvia multicaulis essential oil nanoemulsion was higher than the free bioactive component (Gharenaghadeh et al., 2017). The enhancement of the antimicrobial activity of essential oils or its isolates loaded in nanoemulsions has been reported to be a promotion of interactions with microbial cell membranes in one or more of the following ways: increase in surface area; impact of emulsifiers on phospholipids found in microbial membranes; controlled release of natural oils from the nanosystem; and increased miscibility in aqueous media (Donsi and Ferrari, 2016; Maurya et al., 2021b). However, it cannot be ruled out that it could be connected to factors like water solubility and nanoemulsion particle size.

## Conclusion

The preparation, characterization, and antibacterial activity of geraniol nanoemulsion (G-NE) was carried out in this manuscript. The self-emulsification method allowed the development of nanoemulsions containing geraniol with adequate physico-chemical characteristics, whose stability remained stable during the 28 days of analysis. According to the information on the antibacterial activity, G-NE shown favorable antibacterial activity against the four foodborne pathogenic microorganisms *Staphylococcus aureus*, *Escherichia coli*, *Salmonella typhimurium*, and *Listeria monocytogenes*. Therefore, G-NE, from the tests performed, presented good kinetic stability and efficacy, and could be considered as an alternative to traditional antimicrobial agents to safeguard food and beverages in the long term. In addition, it is necessary to evaluate its practical application in food preservation, since it is important to establish how the interaction with food components will occur in food preservation and safety.

## Data availability statement

The original contributions presented in this study are included in the article/supplementary material, further inquiries can be directed to the corresponding author.

## Author contributions

XF, QZ, KF, CL, and WH: formal analysis. XF, QZ, WT, WZ, SL, and WH: funding acquisition. XF, KF, CL, SL, and WH: investigation. WT, XF, KF, YL, and WH: methodology. QZ, WT, XF, WZ, and YL: project administration. XF, QZ, and WT: resources. XF and QZ: software. XF and WH: supervision. XF: writing – original draft. All authors contributed to the article and approved the submitted version.

## References

[B1] Amrutha B. Sundar K. Shetty P. H. (2017). Spice oil nanoemulsions: Potential natural inhibitors against pathogenic *E. coli* and *Salmonella* spp. from fresh fruits and vegetables. *Lwt Food Sci. Technol.* 79 152–159. 10.1016/j.lwt.2017.01.031 PMC5380633 28416858

[B2] Aswathanarayan J. B. Vittal R. R. (2019). Nanoemulsions and their potential applications in food industry. *Front. Sustain. Food Syst.* 3:95. 10.3389/fsufs.2019.00095

[B3] Babukumar S. Vinothkumar V. Sankaranarayanan C. Srinivasan S. (2017). Geraniol, a natural monoterpene, ameliorates hyperglycemia by attenuating the key enzymes of carbohydrate metabolism in streptozotocin-induced diabetic rats. *Pharm. Biol.* 55 1442–1449. 10.1080/13880209.2017.1301494 28330423 PMC6130491

[B4] Ben Jema M. Falleh H. Serairi R. Neves M. Snoussi M. Isoda H. (2018). Nanoencapsulated *Thymus capitatus* essential oil as natural preservative. *Innov. Food Sci. Emerg. Technol.* 45 92–97. 10.1016/j.ifset.2017.08.017

[B5] Bhattamisra S. Kuean C. Chieh L. Yan V. Lee C. Hooi L. (2018). Antibacterial activity of geraniol in combination with standard antibiotics against *Staphylococcus aureus*, *Escherichia coli* and *Helicobacter pylori*. *Nat. Prod. Commun.* 13 791–793.

[B6] Calvo H. Marco P. Blanco D. Oria R. Venturini M. (2017). Potential of a new strain of *Bacillus amyloliquefaciens* BUZ-14 as a biocontrol agent of postharvest fruit diseases. *Food Microbiol.* 63 101–110. 10.1016/j.fm.2016.11.004 28040156

[B7] Carnesecchi S. Schneider Y. Ceraline J. Duranton B. Gosse F. Seiler N. (2001). Geraniol, a component of plant essential oils, inhibits growth and polyamine biosynthesis in human colon cancer cells. *J. Pharmacol. Exp. Ther.* 298 197–200. 11408542

[B8] Chang Y. McClements D. (2014). Optimization of orange oil nanoemulsion formation by isothermal low-energy methods: Influence of the oil phase, surfactant, and temperature. *J. Agric. Food Chem.* 62 2306–2312. 10.1021/jf500160y 24564878

[B9] Chang Y. McLandsborough L. McClements D. J. (2013). Physicochemical properties and antimicrobial efficacy of carvacrol nanoemulsions formed by spontaneous emulsification. *J. Agric. Food Chem.* 61 8906–8913. 10.1021/jf402147p 23998790

[B10] Chaurasiya C. Gupta J. Kumar S. (2021). Herbal nanoemulsion in topical drug delivery and skin disorders: Green approach. *J. Rep. Pharm. Sci.* 10 171–181. 10.4103/jrptps.JRPTPS_64_20

[B11] Chen W. Viljoen A. M. (2010). Geraniol – a review of a commercially important fragrance material. *South Afr. J. Bot.* 76 643–651. 10.1016/j.sajb.2010.05.008

[B12] Chouhan S. Sharma K. Guleria S. (2017). Antimicrobial activity of some essential oils-present status and future perspectives. *Medicines (Basel)* 4:58. 10.3390/medicines4030058 28930272 PMC5622393

[B13] Donsi F. Ferrari G. (2016). Essential oil nanoemulsions as antimicrobial agents in food. *J. Biotechnol.* 233 106–120. 10.1016/j.jbiotec.2016.07.005 27416793

[B14] El Azab E. Saleh A. Yousif S. Mazhari B. Abu Alrub H. Elfaki E. (2022). New insights into geraniol’s antihemolytic, anti-inflammatory, antioxidant, and anticoagulant potentials using a combined biological and in silico screening strategy. *Inflammopharmacology* 30 1811–1833. 10.1007/s10787-022-01039-2 35932440

[B15] Falleh H. Ben Jemaa M. Neves M. Isoda H. Nakajima M. Ksouri R. (2021). Formulation, physicochemical characterization, and anti- *E. coli* activity of food-grade nanoemulsions incorporating clove, cinnamon, and lavender essential oils. *Food Chem.* 359:129963. 10.1016/j.foodchem.2021.129963 33951609

[B16] Falleh H. Ben Jemaa M. Saada M. Ksouri R. (2020). Essential oils: A promising eco-friendly food preservative. *Food Chem.* 330:127268. 10.1016/j.foodchem.2020.127268 32540519

[B17] Gharenaghadeh S. Karimi N. Forghani S. Nourazarian M. Gharehnaghadeh S. Jabbari V. (2017). Application of *Salvia multicaulis* essential oil-containing nanoemulsion against food-borne pathogens. *Food Biosci.* 19 128–133. 10.1016/j.fbio.2017.07.003

[B18] Grit M. Crommelin D. J. (1993). Chemical stability of liposomes: Implications for their physical stability. *Chem. Phys. Lipids* 64 3–18. 10.1016/0009-3084(93)90053-6 8242840

[B19] Hassan S. T. S. Majerova M. Sudomova M. Berchova K. (2015). [Antibacterial activity of natural compounds – essential oils]. *Ceska Slov. Farm.* 64 243–253. 26841699

[B20] He Z. Zeng W. Zhu X. Zhao H. Lu Y. Lu Z. (2017). Influence of surfactin on physical and oxidative stability of microemulsions with docosahexaenoic acid. *Colloids Surf. B Biointerfaces* 151 232–239. 10.1016/j.colsurfb.2016.12.026 28013167

[B21] Jimenez M. Dominguez J. A. Pascual-Pineda L. A. Azuara E. Beristain C. I. (2018). Elaboration and characterization of O/W cinnamon (*Cinnamomum zeylanicum*) and black pepper (*Piper nigrum*) emulsions. *Food Hydrocoll.* 77 902–910. 10.1016/j.foodhyd.2017.11.037

[B22] Jin X. Sun J. Miao X. Liu G. Zhong D. (2013). Inhibitory effect of geraniol in combination with gemcitabine on proliferation of BXPC-3 human pancreatic cancer cells. *J. Int. Med. Res.* 41 993–1001. 10.1177/0300060513480919 23801065

[B23] Ju J. Xu X. Xie Y. Guo Y. Cheng Y. Qian H. (2018). Inhibitory effects of cinnamon and clove essential oils on mold growth on baked foods. *Food Chem.* 240 850–855. 10.1016/j.foodchem.2017.07.120 28946351

[B24] Khan A. W. Kotta S. Ansari S. H. Sharma R. K. Ali J. (2015). Self-nanoemulsifying drug delivery system (SNEDDS) of the poorly water-soluble grapefruit flavonoid naringenin: Design, characterization, in vitro and in vivo evaluation. *Drug Deliv.* 22 552–561. 10.3109/10717544.2013.878003 24512268

[B25] Kotan R. Kordali S. Cakir A. (2007). Screening of antibacterial activities of twenty-one oxygenated monoterpenes. *Z. Naturforsch. C J. Biosci.* 62 507–513. 10.1515/znc-2007-7-808 17913064

[B26] Li Y. Erhunmwunsee F. Liu M. Yang K. Zheng W. Tian J. (2022). Antimicrobial mechanisms of spice essential oils and application in food industry. *Food Chem.* 382:132312. 10.1016/j.foodchem.2022.132312 35158267

[B27] Li Y. Zhang Z. Yuan Q. Liang H. Vriesekoop F. (2013). Process optimization and stability of D-limonene nanoemulsions prepared by catastrophic phase inversion method. *J. Food Eng.* 119 419–424. 10.1016/j.jfoodeng.2013.06.001

[B28] Liu Q. Huang H. Chen H. Lin J. Wang Q. (2019). Food-grade nanoemulsions: Preparation, stability and application in encapsulation of bioactive compounds. *Molecules* 24:4242. 10.3390/molecules24234242 31766473 PMC6930561

[B29] Marhamati M. Ranjbar G. Rezaie M. (2021). Effects of emulsifiers on the physicochemical stability of oil-in-water nanoemulsions: A critical review. *J. Mol. Liq.* 340:117218. 10.1016/j.molliq.2021.117218

[B30] Marzuki N. H. C. Wahab R. A. Hamid M. A. (2019). An overview of nanoemulsion: Concepts of development and cosmeceutical applications. *Biotechnol. Biotechnol. Equip.* 33 779–797. 10.1080/13102818.2019.1620124

[B31] Maurya A. Prasad J. Das S. Dwivedy A. (2021a). Essential oils and their application in food safety. *Front. Sustain. Food Syst.* 5:653420. 10.3389/fsufs.2021.653420

[B32] Maurya A. Singh V. Das S. Prasad J. Kedia A. Upadhyay N. (2021b). Essential oil nanoemulsion as eco-friendly and safe preservative: Bioefficacy against microbial food deterioration and toxin secretion, mode of action, and future opportunities. *Front. Microbiol.* 12:751062. 10.3389/fmicb.2021.751062 34912311 PMC8667777

[B33] McClements D. J. (2012). Nanoemulsions versus microemulsions: Terminology, differences, and similarities. *Soft Matter* 8 1719–1729. 10.1039/c2sm06903b

[B34] Miladinovic D. Ilic B. Kocic B. Miladinovic M. (2014). An in vitro antibacterial study of savory essential oil and geraniol in combination with standard antimicrobials. *Nat. Prod. Commun.* 9 1629–1632. 25532298

[B35] Mutlu-Ingok A. Devecioglu D. Dikmetas D. N. Karbancioglu-Guler F. Capanoglu E. (2020). Antibacterial, antifungal, antimycotoxigenic, and antioxidant activities of essential oils: An updated review. *Molecules* 25:4711. 10.3390/molecules25204711 33066611 PMC7587387

[B36] Niculau E. Alves P. Nogueira P. Moraes V. Matos A. Bernardo A. (2013). Insecticidal activity of essential oils of *Pelargonium graveolens* l’Herit and *Lippia alba* (Mill) N. E. Brown against *Spodoptera frugiperda* (J. E. Smith). *Quim. Nova* 36 1391–1394.

[B37] Nisar R. Batool Z. Inamullah A. Ghalib A. Nisar H. Emad S. (2022). Dose-dependent alteration of neurobehavioral activities by geraniol a component of essential oil: A study in rats. *Pak. J. Pharm. Sci.* 35 239–245. 10.36721/PJPS.2022.35.1.SUP.239-245.1 35228183

[B38] Noori S. Zeynali F. Almasi H. (2018). Antimicrobial and antioxidant efficiency of nanoemulsion-based edible coating containing ginger (*Zingiber officinale*) essential oil and its effect on safety and quality attributes of chicken breast fillets. *Food Control* 84 312–320. 10.1016/j.foodcont.2017.08.015

[B39] Ortiz N. Jimenez M. Chaverri C. Ciccio J. Diaz C. (2022). Effect on cell growth, viability and migration of geraniol and geraniol-containing essential oil from *Lippia alba* (Verbenaceae) on gastric carcinoma cells. *J. Essent. Oil Res.* 34 65–76. 10.1080/10412905.2021.1975576

[B40] Pavoni L. Perinelli D. R. Bonacucina G. Cespi M. Palmieri G. F. (2020). An overview of micro- and nanoemulsions as vehicles for essential oils: Formulation, preparation and stability. *Nanomaterials* 10:135. 10.3390/nano10010135 31940900 PMC7023169

[B41] Pilong P. Chuesiang P. Mishra D. K. Siripatrawan U. (2022). Characteristics and antimicrobial activity of microfluidized clove essential oil nanoemulsion optimized using response surface methodology. *J. Food Process. Preserv.* 10.1111/jfpp.16886

[B42] Qian C. Decker E. A. Xiao H. McClements D. J. (2012). Physical and chemical stability of beta-carotene-enriched nanoemulsions: Influence of pH, ionic strength, temperature, and emulsifier type. *Food Chem.* 132 1221–1229. 10.1016/j.foodchem.2011.11.091 29243604

[B43] Rao J. Chen B. McClements D. J. (2019). “Improving the efficacy of essential oils as antimicrobials in foods: Mechanisms of action,” in *Annual review of food science and technology*, Vol. 10 eds Doyle M. McClements D. (Palo Alto, CA: Annual Reviews Inc), 365–387. 10.1146/annurev-food-032818-121727 30653350

[B44] Shaker A. Ali M. Fathy H. Marrez D. (2022). Food Preservation: Comprehensive overview of techniques, applications and hazards. *Egypt. J. Chem.* 65 347–363. 10.21608/EJCHEM.2022.110711.5043

[B45] Silva H. D. Cerqueira M. A. Vicente A. A. (2012). Nanoemulsions for food applications: Development and characterization. *Food Bioprocess Technol.* 5 854–867. 10.1007/s11947-011-0683-7

[B46] Siraj A. Naqash F. Shah M. A. Fayaz S. Majid D. Dar B. N. (2021). Nanoemulsions: Formation, stability and an account of dietary polyphenol encapsulation. *Int. J. Food Sci. Technol.* 56 4193–4205. 10.1111/ijfs.15228

[B47] Sneha K. Kumar A. (2022). Nanoemulsions: Techniques for the preparation and the recent advances in their food applications. *Innov. Food Sci. Emerg. Technol.* 76: 102914. 10.1016/j.ifset.2021.102914

[B48] Tadros T. Izquierdo R. Esquena J. Solans C. (2004). Formation and stability of nano-emulsions. *Adv. Colloid Interface Sci.* 108 303–318. 10.1016/j.cis.2003.10.023 15072948

[B49] Teixeira M. C. Severino P. Andreani T. Boonme P. Santini A. Silva A. M. (2017). D-alpha-tocopherol nanoemulsions: Size properties, rheological behavior, surface tension, osmolarity and cytotoxicity. *Saudi Pharma. J.* 25 231–235. 10.1016/j.jsps.2016.06.004 28344473 PMC5355551

[B50] Teo B. S. X. Basri M. Zakaria M. R. S. Salleh A. B. Rahman R. N. Z. R. A. Rahman M. B. A. (2010). A potential tocopherol acetate loaded palm oil esters-in-water nanoemulsions for nanocosmeceuticals. *J. Nanobiotechnol.* 8:4. 10.1186/1477-3155-8-4 20178581 PMC2839968

[B51] Verma K. Tarafdar A. Mishra V. Dilbaghi N. Kondepudi K. K. Badgujar P. C. (2021). Nanoencapsulated curcumin emulsion utilizing milk cream as a potential vehicle by microfluidization: Bioaccessibility, cytotoxicity and physico-functional properties. *Food Res. Int.* 148:110611. 10.1016/j.foodres.2021.110611 34507755

[B52] Zhang Y. Zhong Q. (2020). Physical and antimicrobial properties of neutral nanoemulsions self-assembled from alkaline thyme oil and sodium caseinate mixtures. *Int. J. Biol. Macromol.* 148 1046–1052. 10.1016/j.ijbiomac.2020.01.233 31982537

[B53] Zhuang H. Jiang X. Wu S. Li X. Yan H. (2022). Construction, stability and photodynamic germicidal efficacy of curcumin nanoemulsion stabilised with Maillard conjugate of Wpi-Rha. *Int. J. Food Sci. Technol.* 57 1609–1620. 10.1111/ijfs.15523

